# RNA Back and Forth: Looking through Ribozyme and Viroid Motifs

**DOI:** 10.3390/v11030283

**Published:** 2019-03-21

**Authors:** Marie-Christine Maurel, Fabrice Leclerc, Jacques Vergne, Giuseppe Zaccai

**Affiliations:** 1 Sorbonne Université, Museum National d’Histoire Naturelle, CNRS MNHN UMR 7205, Institut de Systématique, Evolution, Biodiversité, ISYEB, F-75005 Paris, France; jvergne@mnhn.fr; 2 Institute for Integrative Biology of the Cell (I2BC), CNRS, CEA, Université Paris Sud, F-91198 Gif-sur-Yvette, France; 3 Institut de Biologie Structurale CNRS-CEA-UGA, F-380447 Grenoble, France, and Institut Laue Langevin, 71 Avenue des Martyrs, F-38042 Grenoble, France; zaccai@ill.fr

**Keywords:** RNA world, viroid, ribozyme, origins of life

## Abstract

Current cellular facts allow us to follow the link from chemical to biochemical metabolites, from the ancient to the modern world. In this context, the “RNA world” hypothesis proposes that early in the evolution of life, the ribozyme was responsible for the storage and transfer of genetic information and for the catalysis of biochemical reactions. Accordingly, the hammerhead ribozyme (HHR) and the hairpin ribozyme belong to a family of endonucleolytic RNAs performing self-cleavage that might occur during replication. Furthermore, regarding the widespread occurrence of HHRs in several genomes of modern organisms (from mammals to small parasites and elsewhere), these small ribozymes have been regarded as living fossils of a primitive RNA world. They fold into 3D structures that generally require long-range intramolecular interactions to adopt the catalytically active conformation under specific physicochemical conditions. By studying viroids as plausible remains of ancient RNA, we recently demonstrated that they replicate in non-specific hosts, emphasizing their adaptability to different environments, which enhanced their survival probability over the ages. All these results exemplify ubiquitous features of life. Those are the structural and functional versatility of small RNAs, ribozymes, and viroids, as well as their diversity and adaptability to various extreme conditions. All these traits must have originated in early life to generate novel RNA populations.

## 1. Introduction

### 1.1. The RNA World

We know today that RNA is present in all living organisms, in which it performs a variety of structural and metabolic functions. A truly modern “RNA world” exists in each cell, with RNAs in various forms, short and long fragments, single and double-stranded, endowed with multiple roles, informational, catalytic, structural, and regulatory (98% of the human genome is non-coding, transcribed almost entirely, acting as sensors, traveling between organisms, etc). Certain RNA molecules are even capable of carrying out several of these functions.

The “RNA World” hypothesis as an early step in the history of life was so-named by Walter Gilbert [1] after being proposed in the preceding decades by Carl Woese, Francis Crick and Leslie Orgel [2,3,4]. In its formulation [1], the RNA World is partly based on the discoveries of the first ribozymes by Cech and by Altman: the self-splicing RNAs [5] and the RNase P [6], respectively. Further support for the hypothesis was provided by the later discovery of small endonucleolytic ribozymes such as hairpin, HDV, or hammerhead. Only hairpin and hammerhead catalytic motifs are naturally found within plant satellite RNA viruses and viroids [7,8], which perform reversible self-cleavage reactions involved in processing and replication of small circular RNA species [9,10]. Although the HDV ribozyme is not found in viroids, the so called HDV-like ribozymes are also reported in a variety of organisms [11,12,13], several of them are found active in vitro [12]; some of them correspond to mobile elements [14]. Due to its very small size and structural features, the HDV RNA is deemed to be related to viroids [15,16,17,18,19,20,21]. From these perspectives, they are similar to the hammerhead ribozymes which are also found in retrotransposons [22,23]. The hammerhead RNAs are found in other genomes as well [24,25,26,27,28] and may accomplish other biological functions disconnected from their original role in the biology of viroids. For example, hammerhead ribozyme (HHR) motifs were found in the haloarchaeal tailed viruses [28,29]. Although these new biological functions have to be identified, it was shown that they may affect gene expression to different extents depending on the genomic and genetic contexts [30]. Their catalytic efficiency also depends on their sequence and fold [31].

In the past five years, several studies reported the self-assembly of natural ribozymes leading to dimers that modulate the overall catalytic efficiency [22,32,33]. This ability for self-dimerization also appears in artificially selected variants [34] or (re)design ribozymes [35]. It was shown recently that it also applies to the Avocado Sun Blotch Viroid (ABSVd) [33].

The RNA World hypothesis raises important questions. Could RNA have existed alone? Is there a fossil trail of ancient RNAs? Diener’s hypothesis, proposed in 1989 [36] and re-discussed recently [37,38], enumerates the pros and cons in considering viroids as relics from the RNA world. So, we will not address all the arguments raised for or against this hypothesis. Instead, we will focus on two intrinsic properties of viroids and/or ribozymes that may keep the discussion open about viroids and their evolution: i.e., the self-assembly and replication ability in other kingdoms. Our recent results on these two topics with the Avocado Sun Blotch Viroid (ASBVd) will be presented as illustrations.

### 1.2. Viroids and Ribozymes

Viroids are the smallest plant pathogens. From 300 to 500 ribonucleotides long, the single-stranded RNA molecules can pair and form loops and bulges. They were discovered in 1971 by Theodor Diener [39] following the observation of potato tubercle disease, which was first attributed to bacterial infection, then to a virus, then to viroids. Viroids are absolutely «free» in nature. As opposed to viruses, they are composed of free RNA without envelope or capsid and do not code for any protein. Some viroid structures are similar to those of other RNAs from which they might have emerged; for example, ASBVd and transfer RNA [40,41] or small plant circular RNA [42], some of which correspond to mobile elements [23,43]. Viroids are circular, rod-like, or flexible, the existence of several different motifs enhancing their probability of survival. They replicate in either the cell nucleus or chloroplast, suggesting diversity and adaptability to different environments. Two viroid families are known today, the Pospiviroidae including 5 genera (pospiviroids, hostuviroids, cocadviroids, apscaviroids, and coleviroids) and 27 species, and the Avsunviroidae with three genera (avsunviroids, pelamoviroides, and elaviroides), and 4 species. Pospiviroidae replicates in the cell nucleus by the asymmetric rolling circle mechanism and in chloroplasts for Avsunviroidae by the symmetrical rolling circle mechanism which implicates a hammerhead ribozyme (HHR). In this symmetric variant of the rolling circle mechanism, the monomeric (+) circular RNA serves as the template for an RNA polymerase that replication leads to a linear (−) oligomer. This RNA self-cleaves through a hammerhead ribozyme from RNA itself. The monomeric (−) linear RNA is then ligated to the circular form that serves as the template for synthesis of the linear (+) oligomer, which after self-cleavage and circularization gives the final product that is the monomeric (+) circular RNA [44,45,46]. The mechanistic aspects of the cleavage reaction in the hammerhead motifs is as follows: initiation by a nucleophilic attack of the 2${}^{\prime }$-hydroxyl group on the adjacent phosphorus. The reaction mechanism is SN2, with a trigonal bipyramidal transition state. The departure of the 5${}^{\prime }$-oxyanion leaves a 2${}^{\prime }$-3${}^{\prime }$ cyclic phosphate. In physiological conditions, the transesterification reaction involves divalent metal ions as cofactors and is accelerated by general acid/base catalysis and by conformational effects facilitating the formation of the in-line geometry [47,48,49,50]. The ligation is the exact reverse of the cleavage reaction [51].

### 1.3. RNA Dimerization: Bonded/Non-Bonded Dimers

There is an apparent antagonism between two essential properties which are expected for RNAs as primordial molecules from the RNA World: e.g., acting as a template for replication and carrying a catalytic activity that would require a stable fold. One way to escape from this paradox between a stable fold but a poor template (catalytic activity) or a poorly folded RNA and a good template (templating ability) [38] was proposed. It is based on G:U wobble pairing and the fold asymmetry between the strands (+) and (−), making one strand more prone to catalysis and the other one more prone to templating [52]. One may imagine an alternative strategy to resolve this dilemma coming from the ability of some viroids to form bonded dimers or so-called “double-hammerhead” structures.

#### 1.3.1. Bonded Dimers or Double-Hammerhead Structures

In viroids, some bonded dimeric structures are generated as a result of the fusion of two “single-hammerhead” structures through the merging of both stems III. This typically occurs in the oligomeric forms, corresponding to the (+) or (−) strands, which undergo self-cleavage to generate linear monomers. Such viroids with double-hammerhead structures were reported for a long time in ASBVd [53], but more recent works confirm it is also a feature shared with other viroid-like RNAs such as the rice yellow mottle sobemovirus (RYMV) [54], the satellite cereal yellow dwarf virus-RPV (satRPV) [55], or other unrelated RNAs like the Penelope-like retrotransposons [22].

**Figure 1:**
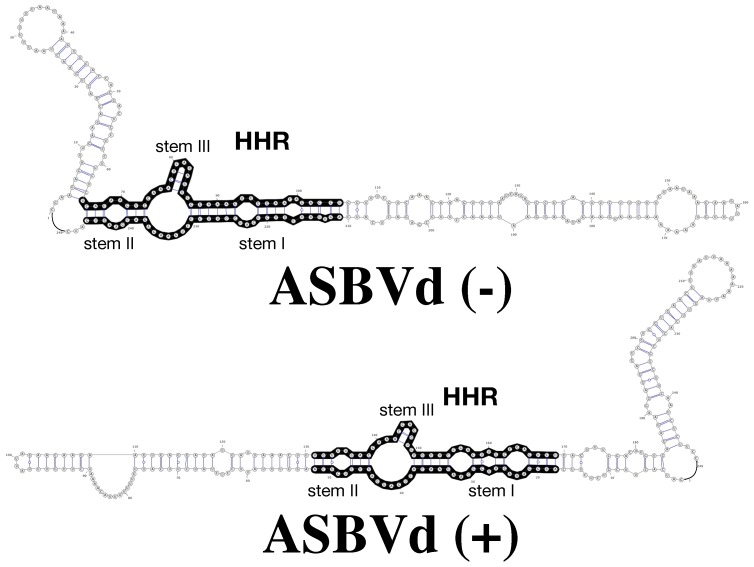
RNA 2D structures of Avocado Sun Blotch Viroid (ABSVd) (−) and ASBVd (+) (top and bottom, respectively). The full length genome of ASBVd can fold into 2D structures that preserve the hammerhead ribozyme (HHR) motif (regions in black) in both the (−) and (+) strands; the HHR motif of ASBVd (−) is more stable, with 3 base-pairs in stem III but only two base-pairs in ASBVd (+).

Both strands of viroid in the family Avsunviroidae and similar viroid-like RNAs are by definition self-complementary, but they still maintain a sequence which is compatible with a HHR fold on both strands (+) and (−) (Figure 1). However, the two corresponding HHR motifs are not equivalent; in all the above-mentioned examples, the HHR fold on either strand is unstable with a very short stem III stem as in ASBVd (+) with two base-pairs or less (Figure 1). Actually, the double-hammerhead structures are needed to maintain the catalytic activity [53]. In other cases, like in the peach latent mosaic viroid (PLMVd), the stem III is long enough and stable, and no double-hammerhead structure is detected [56].

From the current literature, we can classify the viroids and viroid-like RNAs based on three profiles: (1) the “bad catalysts” (inactive) with an unstable HHR fold for both strands (+) and (−) which rely on the formation of double-hammerhead structures for the calytic activity; (2) the “good catalysts” (highly efficient) with a stable HHR fold for both strands (+) and (−); (3) the “intermediate catalysts” with a differential catalytic efficiency between the two strands: an active strand (−) as a single-hammerhead structure and an inactive strand (+) that becomes active only as a double-hammerhead structure. The first profile includes: the satellite RNA of cereal yellow dwarf virus-RPV (satRPV RNA) [55,57], the small circular RNA (sc-RNA) associated with rice yellow mottle sobemovirus (RYMV), the Newt satellite RNA [53], etc.; the second one: the peach latent mosaic viroid (PLMVd) [56], the Eggplant latent viroid (ELVd) [58], the circular satellite RNA (sat-RNA) associated with the lucerne transient streak sobemovirus (LTSV) [30,54], the Apple hammerhead viroid-like RNA (AHVd) [59], the Chrysanthemum chlorotic mottle viroid (CChMVd) [30], etc.; the third one: the small circular RNA associated with a stunting syndrome incarnations (CarSV) [42], the Avocado Sun Blotch Viroid (ASBVd) [53,60], etc.

In the RNA World, the dimerization through the formation of double-hammerhead structures could be seen as a strategy to resolve the “biocatalyst/template” conflict, with viroid-like RNAs as good templates for replication and sub-optimal catalysts. By harboring unstable HHR folds, they remain good templates; by merging together inactive single-hammerhead structures into active double-hammerhead structures, they maintain a required catalytic activity. Moreover, the ability to form such bonded dimers probably offered a selective advantage against degradation in a clay mineral environment close to the RNA World conditions [61].

#### 1.3.2. Non-Bonded Dimers

In ribozymes, another mode of dimerization is observed through a reversible self-assembly mechanism. In contrast with the double-hammerhead structures, the two monomers are not covalently bonded but associated in a dimer through weak non-bonded interactions. In this kind of dimer, a loss of catalytic activity was described in some artificial variants of natural ribozymes because of misfolded RNAs or active sites in the dimer (HDV ribozyme [62], hairpin ribozyme [34]). A particular case of unusual dimerization was also reported in the Varkud satellite (VS) ribozyme: a dimer is formed by a strand exchange between two monomers, leading to a more complex and catalytically active ribozyme [32]. This mode of dimerization could have facilitated the emergence of new and more complex RNA folds [32]; it could be regarded as a potential trait from the RNA World. However, non-bonded viroid dimers had not been reported until recently and only in the case of ASBVd [33].

### 1.4. Small Angle Neutron Scattering (SANS) and the Study of Multimeric Complexes

Small angle neutron scattering (SANS) is a well adapted method to study fragile nucleic acid structures and interactions in solution [63,64]. The sample solution of the order of mg/mL is examined in a quartz cell, where solvent conditions and/or temperature can be easily changed, in-beam. There is no radiation damage to the sample even when irradiated for a long time at high temperature. SANS data in the Guinier approximation are interpreted, on an absolute scale, in terms of low resolution mean structural parameters for the particles in the solution [63,64]. From I(0), the forward scattered intensity, is obtained the number-average molecular weight of the particles (distinguishing monomers from dimers, for example); from the angular dependence of the intensity, are obtained particle radii of gyration and cross-sectional radii of gyration for rod-like configurations.

Bi-molecular RNA interactions such as linear-linear, loop-loop, loop-linear, or kissing interactions are important in the control of biological activity, and hairpin loops present rich potential for establishing both intra- and inter-molecular interactions through standard Watson–Crick base pairing or non-canonical interactions. In a previous study, we applied SANS with native gel electrophoresis and analytical centrifugation to the structures and self-interactions of two 85 base adenine-dependent hairpin ribozymes (ADHR1 and ADHR2) [34]. Similar results were obtained for the two. At room temperature, the ribozymes self-associated in structures with a cross-section corresponding to two double strands side-by-side. Non-covalent dimers predominate at low concentration (∼0.1 mg/mL); they associate into longer rods, with increasing concentration (∼1 mg/mL). Above 65 °C, the structures dissociated into compact monomers, with a radius of gyration similar to that of tRNA (about 70 bases). Only monomers were active for catalysis, suggesting that dimer formation, probably by preventing correct loop docking, could act as an inhibition mechanism for the regulation of hairpin ribozyme catalysis.

### 1.5. Viroids in Non-Plant Hosts

Considering the absence of an envelope, the fact that viroids are non-coding and the existence of ribozymes, it is tempting to consider them as vestiges of ancient RNAs as Diener proposed in 1989 [36]. As noted above, the hammerhead RNA motifs appear to be widely distributed in all kingdoms of life. The reason for their widespread distribution is subject to debate. They could correspond to viroid-like RNAs that diverged or unrelated RNAs that just happen to be the result of convergent evolution [38]. Apart from viroids and viroid-like RNAs, functional HHR were also found in plant, the first reported case was in the genome of *Arabidopsis thaliana*. In this particular case, the hypothesis of a viroid origin was discarded based on phylogenetic analyses [65]. Among the nine HHR motifs that were found in humans, the activity for two of them was confirmed experimentally; several of these hits from a particular class of HHR motifs (type II) are actually considered as false positive cases [28]. In other eukaryotes, 4 out of 18 candidates do not have any catalytic activity in vitro but a short stem III with one, two or at most three base-pairs [28] (Figure 1). The formation of double-hammerhead structures was invoked in these 4 cases to explain the absence of catalytic activity in vitro on monomers. As described above, this ability to form bonded dimers is a feature that was primarily identified in viroid-like RNAs and viroids (ASBVd), which is also found in mobile elements in eukaryotes. It also requires a palindromic sequences in the terminal loop of stem III; in eukaryotes, another feature is also needed: a tandem arrangement of both HHR motifs in the genome [22]. Whether this recurrent HHR superstructure is the result of convergent evolution or not is an interesting debate. Both hypotheses of convergent and divergent evolution might be true depending on the part of the tree of life we consider. So, it might be difficult to give a clear answer to this question.

However, one supporting argument to consider viroids as relics of the RNA World is their adaptability to other hosts than plants, non-photosynthetic Eukarya, Bacteria, and even potentially in viruses like the Hepatitis delta virus [15,16,17,18,19,20,21]. This capability may help to elucidate several unexplained symptoms in other species than plants [21]. If viroid or viroid-like RNAs are present in non-plant hosts, they might be difficult to detect unless to analyze in details the transcriptomes of these other potential hosts [66]. Alternatively, their dissemination through the other kingdoms of life may have been hindered by the natural barrier which is the cell membrane. With regard to membrane penetration and trafficking, plant and animal cells have very different characteristics. Usually, the viroid infection involves a transmission by mechanical means that could be facilitated by insects [67].

## 2. ASBVd as a Viroid Model

### 2.1. ASBVd (+)/(−) and the Biocatalyst/Template Paradox

**Figure 2:**
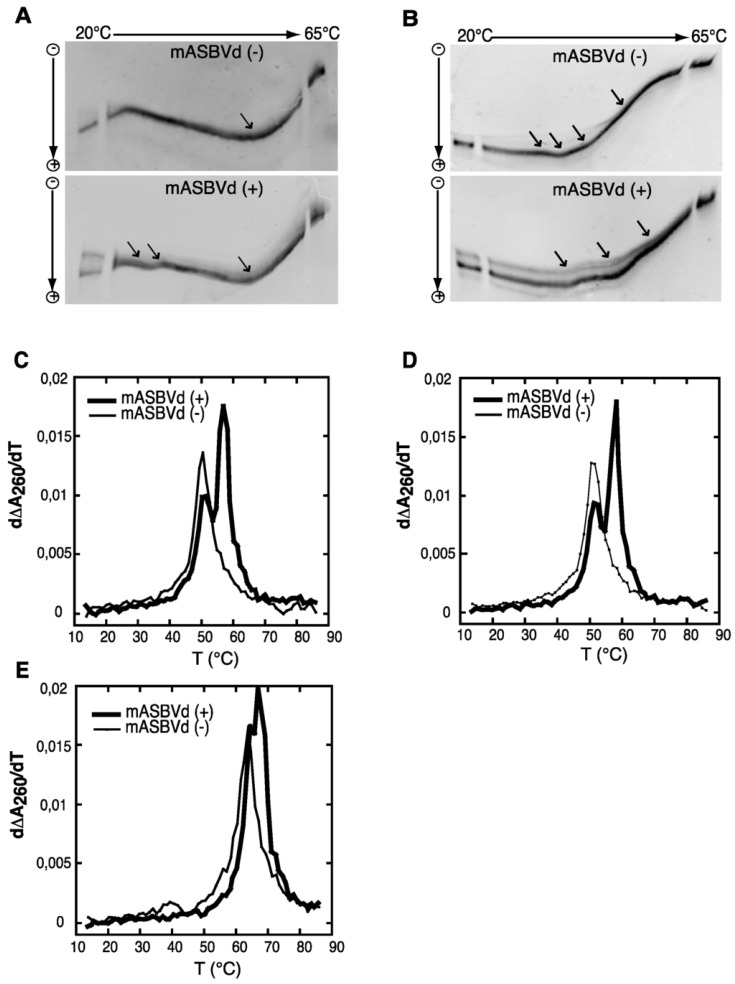
TGGE (Thermal Gradient Gel Electrophoresis) and melting curves of the monomeric forms of ASBVd: mASBVd (+) and mASBVd (−) transcripts. (**A**) TGGE analysis performed by native gel electrophoresis 8% PAGE in 0.2 TBE Buffer. (**B**) TGGE analysis performed by native gel electrophoresis 8% PAGE in 0.2 TBE Buffer containing 20 mM magnesium acetate. Migration was monitored between 20 °C and 65 °C. The arrows denote the transition temperatures. Derivative absorbance melting profile were determined either in 150 mM KCL and 10mM sodium cacodylate (pH 7.2). (**C**) or with in addition, 100 mM MgCl${}_{2}$ (**D**) or 1M NaCl. (**E**) The first derivative profiles are shown with a thick curve for mASBVd (+) and a thin curve for mASBVd (−). The apparatus fixes the temperature gradient, which is transferred from left to right on the figure; the arrows indicate the transition temperatures that correspond to the conformational changes. From Delan-Forino et al. [70].

We have studied the viroid model: Avocado Sun Blotch Viroid (ASBVd), one of the smallest rod-like viroids (247 nts), including a hammerhead ribozyme (HHR) of 42 nucleotides. In vivo, both complementary strands (+) and (−) (harboring a HHR motif: Figure 1) can be used as template for replication once the first replication cycle completed from the infecting single-stranded RNA: ASBVd (+) conventionally designed for the most abundant infectious RNA polarity can be found in different concatenated multimeric forms up to octamers, while ASBVd (−) is just present as a monomer or dimer with a more efficient cleavage activity [45,68]. On native gel and thermal gradient gel electrophoresis (TGGE) [69] one major profile was observed for ASBVd (−) at all temperatures, while ASBVd (+) adopts alternative folds at lower temperatures. The derivative absorbance (dΔA260/dT) of the melting profiles (Figure 2) shows two transitions for ASBVd (+) and one for ASBVd (−) [70]. We conclude there are 2 non-symmetrical polarities (+) and (−) with different structures and different catalytic activities (Figure 1).

### 2.2. Viroid Structures and Interactions Revealed by SANS

**Figure 3:**
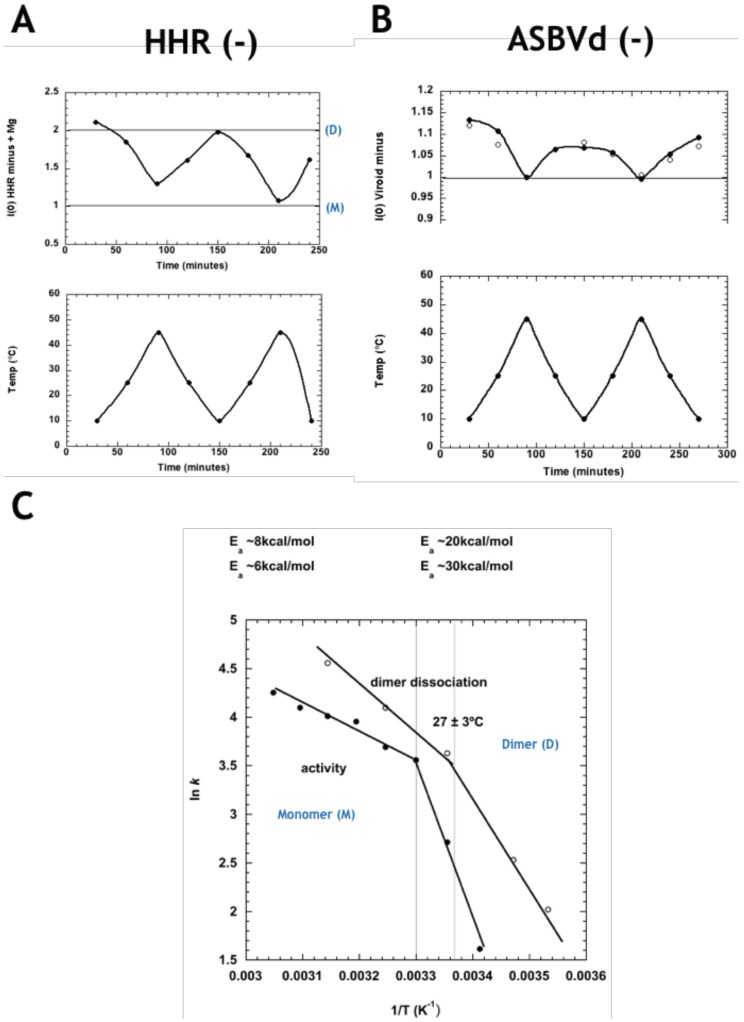
(**A**) Normalized forward scattered small-angle neutron scattering (SANS) intensity for HHR (−) in the presence of Mg++ at the corresponding temperature on the plot below. The values at 1.00 and 2.00 correspond to the intensity expected for the monomer (M) and dimer (D), respectively. (**B**) Normalized forward scattered SANS intensity for ASBVd (−) in presence (filled circles) and absence (open circles) of Mg++, at the corresponding temperature on the plot below. The data indicate a small amount of dimer formation at the lower temperature. (**C**) Arrhenius plots of HHR (−)/Mg rate of dimer dissociation (open circles, calculated from (A), and the ribozyme’s catalytic activity (filled circles), revealing parallel behaviour. Modified from Leclerc et al. [33].

The SANS approach developed in the ADHR study was applied to ASBVd (−) and its derived 79-nt HHR (−). It revealed a reversible temperature-dependent dimer association in both molecules. In Figure 3A, I(0) values (normalized to 1 for the monomer) are seen to oscillate in the region between 1 (100% monomers M, at high temperature) and 2 (100% dimers D, at low temperature). HHR (−)/Mg (in presence of Mg++) displays a striking temperature driven oscillatory behavior, between dimer-dominated scattering at low temperature and monomer-dominated scattering at the higher temperature. The values for ASBVd (−) (Figure 3B) indicated a reversible low level of particle association at low temperature (accounted for by about 5% of the particles forming dimers) with full dissociation to monomers at the higher temperature, both in presence and absence of Mg++. To explore the functional relevance of the SANS observations, the Arrhenius plot calculated from the rate of HHR (−)/Mg dimer dissociation as a function of temperature was compared to a similar plot calculated for catalytic activity of the same ribozyme published previously (El-Murr 2012) (Figure 3C). The two plots are strikingly similar, showing a break at about 27 °C between regimes of activation energy 7 ± 1 kcal/mol and 25 ± 5 kcal/mol, at higher and lower temperatures, respectively, suggesting that the rate-determining step corresponds to the dimer/monomer transition, with dimer dissociation correlated with higher activity.

**Figure 4:**
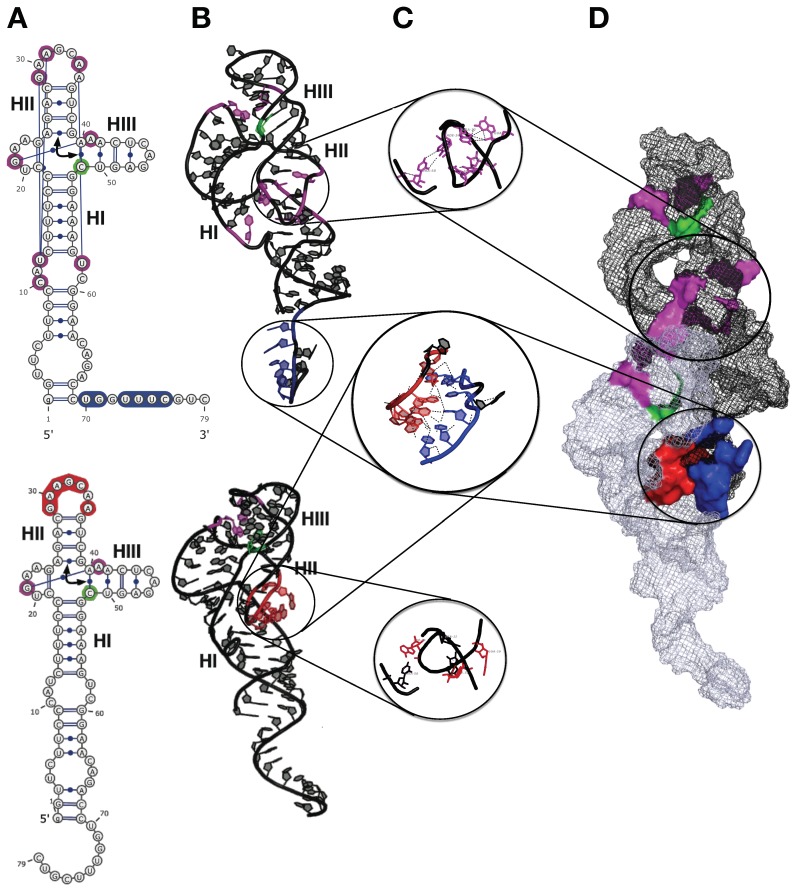
3D Model of self-association for the hammerhead ribozyme (HHR) from ASBVd(−). (**A**) 2D structures of the two monomers as they are in the HHR dimer. (**B**) 3D structures of the two monomers as they are in the HHR dimer. (**C**) Zoom in on the regions involved in the monomer-monomer interactions and in the 3D contacts within the first monomer (top). (**D**) 3D structure of the HHR dimer. The long-range intramolecular contacts are indicated in magenta and the cleavage site in green. The paired regions are blue (first monomer) and red (second monomer). Modified from Leclerc et al. [33].

The 2D and 3D modeling of monomeric and dimeric HHR (−) suggested that the intermolecular contacts stabilizing the dimer (between HI and HII domains) compete with the intramolecular ones, stabilizing the active conformation of the full-length HHR required for an efficient self-cleavage (Figure 4). Similar competing intra- and inter-molecular contacts were proposed in ASBVd (−), though with a remoter region from an extension of the HI domain. The structural basis for the lower catalytic activity in the HHR dimer is due to a competition between the intermolecular contacts responsible for the self-association of the two monomers and the long-range intramolecular contacts that stabilize the active conformation of the HHR monomer. In the dimer complex (Figure 4), the first monomer is expected to be fully active since all the intramolecular contacts are preserved for the RNA to fold into its catalytically active conformation. On the other hand, these intramolecular contacts are abolished in the second monomer because the corresponding residues are involved in the monomer-monomer interaction (Figure 4A). The 3${}^{\prime }$-end from the first monomer interacts with the loop region of the HII domain from the second monomer, thus preventing the second monomer from adopting its active conformation necessary for an efficient catalysis (Figure 4B). This loop region is remodeled during the self-association so that it can pair to the single-stranded 3${}^{\prime }$-end of the first monomer. The resulting paired region corresponding to a half helix turn, and the residues involved in the intramolecular contacts preserved in the first monomer or lost in the second monomer are shown in details in Figure 4C. The dimer adopts a conformation where only the first monomer can be fully stabilized in the active conformation; the active site from the second monomer does not fold properly being destabilized due to the intermolecular contacts formed during the self-association [33] (Figure 4D).

### 2.3. Replication of ASBVd in Other Kingdoms

It remained to be determined whether or not ASBVd can replicate in other types of plastids (e.g., proplastids, amyloplasts, etioplasts, and chromoplasts) and in other species than plants. Following this line of thought we showed an efficient cleavage of ASBVd by the HHR and followed the transcription/replication, as well as circularisation of the viroid in the yeast *S. cerevisiae*. The process was followed for 25 generations that is the persistence of the viroid in *S. cerevisiae*, showing unsuspected functional diversity and adaptability and confirming that the ribozyme can fulfill the cleavage/ligation reaction during the rolling circle replication process in a non-plant host [71].

**Figure 5:**
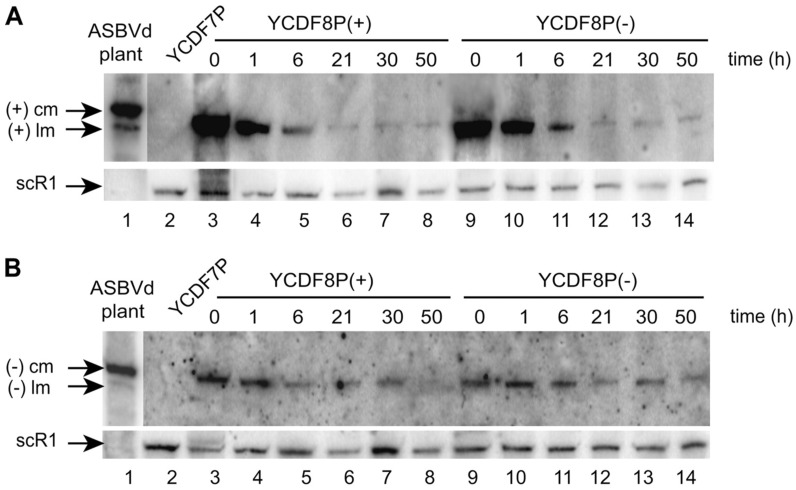
Northern blot analyses (by hybridization using riboprobes corresponding to the replicated ASBVd (−) (**A**) or (+) (**B**)) of the persistence of mASBVd in *S. cerevisiae*. (**A**) mASBVd (+) evidenced by riboprobe (−) and (**B**) mASBVd (−) evidenced by riboprobe (+) (cm: circular monomer; lm: linear monomer). (**A**,**B**) Total RNAs were extracted from the YCDF7P, YCDF8P(+), and YCDF8P(−) strains. From Delan-Forino et al. [71].

The RNAs were extracted at different times from 0 to 50 h and then analyzed by Northern blotting (Figure 5A,B). After 50 h corresponding to about 25 generations, both polarities of the monomeric ASBVd (mASBVd) were still detected (Figure 5A,B, lanes 8 and 14). These results strongly suggest that the plasmid transcription is not mandatory for the maintenance of mASBVd via RNA-RNA replication in yeast, at least during 25 generations. We concluded on the functional diversity and unsuspected adaptability to a non-plant host.

**Figure 6:**
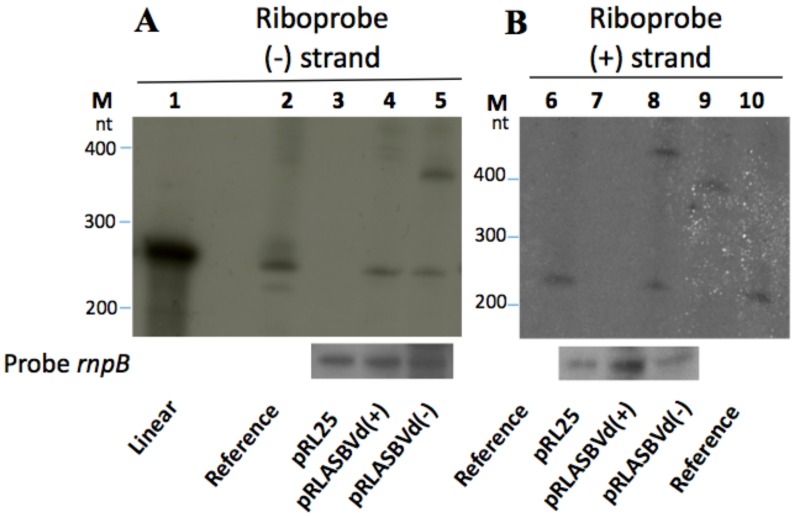
Analysis of ASBVd replication in the filamentous cyanobacteria Anabaena. RNAs extracted from Anabaena harboring either the empty pRL25 plasmid (3,7), pRLASBVd (−) which is the plasmid recombinant ASBVd (−) derived from the pRL25 plasmid (5, 9) or pRLASBVd (+) (4, 8). Northern blot analysis by hybridization using riboprobes corresponding to the replicated ASBVd (−) (**A**) or (+) (**B**). Lane 5: linear replicate (+) detected by riboprobe (−) when RNAs/ pRLASBVd(−) was loaded. Lane 8: linear replicate (−) detected by riboprobe (+) when RNAs /pRLASBVd(+) was loaded. Modified from Latifi et al. [73].

Because ASBVd replicates in plant chloroplasts which have their ancestry in cyanobacteria, via endosymbiosis [72], we asked ourselves if one can detect the presence of viroids in the Bacterial domain, in particular, in cyanobacteria. *Anabaena* (*Anabaena* sp. PCC7120) is a filamentous cyanobacterium that is a photosynthetic prokaryote (blue-green algae). We detected (−) and (+) ASBVd in *Anabaena*, that is linear transcript (+) was detected by riboprobe (−) when RNAs/ pRLASBVd(−) was loaded as well as linear transcript (−) detected by riboprobe (+) when RNA/pRLASBVd(+) was loaded [73] (Figure 6).

The replication of ASBVd in *Anabaena* had no effect on the physiology of this filamentous cyanobacterium. Replication of the viroid in *S. cerevisiae* has also been shown to be asymptomatic [71]. Together, these results raise the question on whether or not various non-infectious RNAs may exist across the Bacteria, Archaea, Eukarya realms where they would replicate via an RNA-RNA mechanism. If so, what are the evolutionary forces that maintained this "RNA World" in parallel to the “DNA World”?

## 3. Conclusions

The higher order of organization through dimerization, i.e., the formation of bonded dimers (double-hammerhead structures), is a particular feature of ASBVd to do more under tight evolutionary constraints for such small genomes that should preserve functional HHR [74,75]. The simultaneous presence of viroids or viroid-like RNAs with different catalytic profiles: from inactive or bad catalysts as single-hammerhead structures to intermediate and good catalysts can be the result of the adaptation process to specific hosts and of the survival of properties which originate from the RNA World. On the other hand, the host range of viroids that is restricted to plants has often been presented as an argument against the hypothesis to consider viroids as relics from the RNA World. However, they are also functional in eukaryotic cells [71,73]. So, we cannot rule out that they have not been found yet in other organisms until they have been searched using the latest technologies applied in RNomics.

ASBVd has adapted to different hosts: it normally replicates in the chloroplast of avocado, but it can also replicate in the cyanobacterium *Anabaena*. We have further showed that ASBVd replicates and persists in *S. cerevisiae*, a non-photosynthetic eukaryote. It is legitimate to speculate on extant viroids as descendants of “free-living” proviroids that invaded ancient cyanobacteria, which would later become endosymbionts, evolving in chloroplasts by usurping the biochemistry of their hosts. Finally, in a “viroids as living fossils” scenario [36,38], we must envisage that polymerase and ligase activities might have been lost by the viroid ancestors since it has been demonstrated that these two activities are possible in vitro. They were indeed discovered in artificially evolved RNAs by SELEX, for instance, in the RNA-polymerase ribozyme (PDB ID: 3IVK) [76] and RNA ligase ribozyme (PDB ID:2OIU) [77]. Viroid-like ancestors from the RNA World would need to carry three catalytic activities: RNA polymerase, RNA ligase, and RNase which is the sole remaining ribozyme activity in the Avsunviroidae family [78].

**Figure 7:**
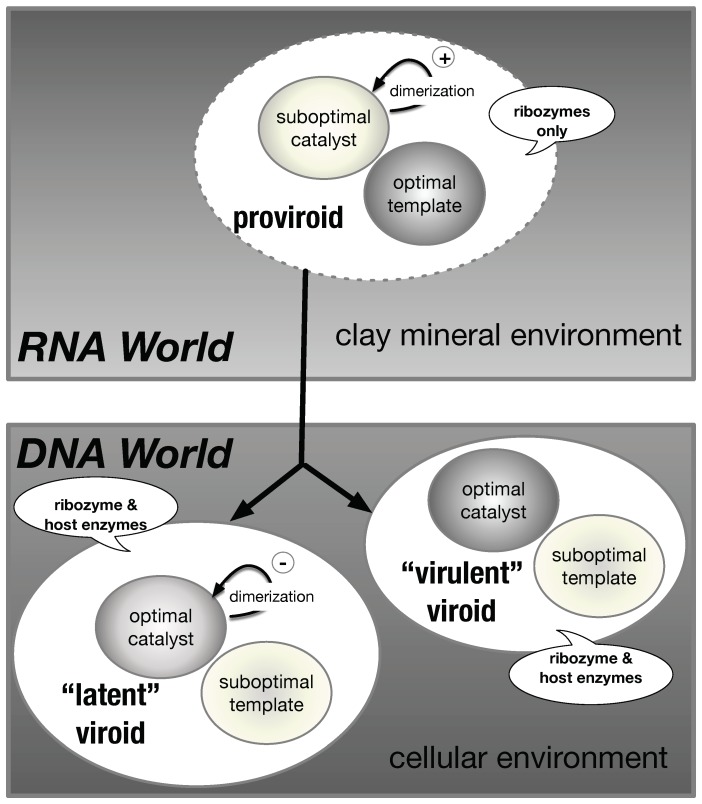
A model for the evolutionary path of the transition from proviroids (RNA World) to viroids (DNA World). In a clay mineral environment (RNA World), the proviroids are suboptimal catalysts that retain an optimal template activity. The catalytic activity can be increased by “bonded” dimerization (double-hammerhead structures). In a host (DNA World), “virulent” viroids are efficient as catalysts and suboptimal templates, while “latent” viroids (ASBVd) have a regulatory control of their catalytic activity by “non-bonded” dimerization (non-bonded dimeric structures). The bubbles indicate the enzymes required for the replication cycle.

Assuming that viroid-like RNAs and viroids have lost two of their initial catalytic activities, we may propose an evolutionary path from the RNA World to the DNA World (Figure 7). The viroids of the Avsunviroidae family still carry an RNAse activity which is necessary for their life cycle. During the transition, the viroids could override the constraints imposed initially by the “biocatalyst/template” paradox. In the RNA World, the proviroids with a “bad catalyst” profile are good templates for replication and preserve the necessary RNase activity through the dimerization into double-hammerhead structures which are catalytically active. In the RNA World, the dimerization would also confer a selective advantage against RNA degradation [61]. Then, slight changes in the sequence of the HHR motif could lead to some intermediate configuration where the catalytic activity is improved in a strand-specific way: the HHR fold is stabilized only on one strand (e.g., by lengthening the stem III), making the viroid more efficient as a catalyst. The opposite strand would keep being a good template with a catalytic activity relying on the formation of bonded dimers. Additional concerted changes in the HHR sequence could result in more stable HHR folds on both strands carrying a highly efficient catalyst. In a cellular environment, the viroids can evolve by a selection for better catalysts, where the ribozyme-dependent RNase activity is optimal while the template can become suboptimal. By using host enzymes for the other catalytic activities, there is no more selective pressure for good templates (Figure 7).

In this hypothetical evolutionary path, ASBVd would correspond to some half-way state where the catalytic efficiency has been partially optimized. The reason it did not evolve towards a fully optimal catalyst found in other viroids may seem puzzling. However, it might be explained eventually by different sources of evolutionary constraints creating antagonistic epistases [75] in order to escape from the plant’s immune response, and to preserve at the same time the HHR folds on both strands and the associated long-distance interactions necessary for catalytic efficiency (Figure 4). Only a few nucleotide substitutions and insertions are sufficient to alter the virulence/latency of ASBVd variants [79]. One may invoke that ASBVd has an extremely narrow host range and thus has co-evolved under host-specific evolutionary constraints. Two other members of the Avsunviroidae family, ChCMVd and PLMVd, also have a narrow host range but they do harbor some stable HHR folds on both strands with high catalytic efficiency. However, these two viroids belong to genus *Pelamoviroid* and adopt complex multi-branched secondary structures which are very different from the simple rod-like structure of ASBVd [80]. Thus, the peculiar secondary structure of ASBVd (only member of the genus Avsunviroid) might be tightly bound to the evolutionary trajectory of this viroid as a special case of “intermediate” catalyst. ASBVd would be the only example of true viroid with a sub-optimal catalyst, since all the other listed “bad catalysts” correspond to viroid-like RNAs. So, it might correspond to a trace for direct inheritance from the RNA World.

The existence of another mode of dimerization in viroids through the self-assembly of monomeric RNAs is also a particularity of ASBVd. As mentioned previously, ASBVd exists in two non-symmetrical polarities (+) and (−) with different structures and catalytic activities. A joined thermodynamic analysis of structural and catalytic data indicates that the rate-determining step corresponds to a dimer/monomer transition. Models suggest that the intermolecular contacts stabilizing the dimer (between HI and HII domains) in HHR (−) compete with the intramolecular ones, stabilizing the active conformation of the full-length HHR required for efficient self-cleavage (Figure 4). Similar competing intra- and inter-molecular contacts are proposed in ASBVd (−), though with a remoter region from an extension of the HI domain. In vivo, ASBVd (+) can be found in different concatenated multimeric forms up to octamers, while ASBVd (−) is just present as a monomer or dimer with a more efficient cleavage activity. Each polarity could play a distinct role during the viroid life cycle. Lowering the temperature-dependent catalytic activity of ASBVd (−) might play a regulatory role associated with the day/night cycle. Alternatively, it could be a mechanism aimed at synchronizing the transcription of both (+) and (−) strands. The dimerization through weak non-bonded interactions could attenuate the virulence and allow the viroid to co-evolve within its hosts by switching between phases of virulence or latency (Figure 7). Such a mechanism is already in action with the apparition of ASBVd variants responsible for a transition to a milder form of infection [79]. This ability to form non-bonded dimers could result from some recent event since it is only reported for ASBVd to date.

The Avocado Sun Blotch Viroid (ASBVd) is unique since it combines “modern” and “ancient” features that may be attributed to either the RNA or DNA World. Proviroids would have been good templates for replication but poor catalysts (biocatalyst/template paradox). As a sub-optimal catalyst, ASBVd is reminiscent of those proviroids whose dimerization through double-hammerhead structures would have protected them from degradation in a clay mineral environment. ASBVd still has this ability to form such dimers, a trait which is also found in many mobile elements or viroid-like RNAs harboring a HHR motif but absent from the other known viroids of the Avsunviroidae family. On the other hand, the formation of non-bonded dimers, which is unique to ASBVd, tends to decrease the catalytic activity; it could be a recent adaptation and the sign of co-evolution in the DNA World.

## Abbreviations

The following abbreviations are used in this manuscript:

ASBVd	Avocado Sun Blotch Viroid
mASBVd	monomeric ASBVd
pRLASBVd	plasmid recombinant ASBVd derived from the pRL25 plasmid
HHR	Hammerhead ribozyme
TGGE	Temperature gradient gel electrophoresis
ADHR	adenine-dependent hairpin ribozyme
SANS	small-angle neutron scattering
